# 5-aminolaevulinic acid (5-ALA) accumulates in GIST-T1 cells and photodynamic diagnosis using 5-ALA identifies gastrointestinal stromal tumors (GISTs) in xenograft tumor models

**DOI:** 10.1371/journal.pone.0249650

**Published:** 2021-04-07

**Authors:** Makiko Sasaki, Mamoru Tanaka, Hiroshi Ichikawa, Taketo Suzuki, Hirotada Nishie, Keiji Ozeki, Takaya Shimura, Eiji Kubota, Satoshi Tanida, Hiromi Kataoka

**Affiliations:** Department of Gastroenterology and Metabolism, Nagoya City University Graduate School of Medical Science, Nagoya, Aichi, Japan; Massachusetts General Hospital, UNITED STATES

## Abstract

Gastrointestinal stromal tumor (GIST) diagnosis using conventional gastrointestinal endoscopy is difficult because such malignancies cannot be distinguished from other types of submucosal tumors. Photodynamic diagnosis (PDD) is based on the preferential uptake of photosensitizers by tumor tissues and its detection by fluorescence emission upon laser excitation. In this study, we investigated whether PDD using 5-aminolevulinic acid (5-ALA), a standard photosensitizer used worldwide, could be used for GIST diagnosis. 5-ALA is metabolized to endogenous fluorescent protoporphyrin IX (PpIX). We examined the accumulation of PpIX in GIST-T1 cells using flow cytometry and immunofluorescent staining. Furthermore, we established GIST-T1 xenograft mouse models and examined PpIX accumulation in the resultant tumors. PpIX accumulated in GIST-T1 cells and was localized mainly to lysosomes. PpIX accumulation was also observed in murine xenograft tumors. Moreover, tumor and normal tissues could be distinctly identified by relative PpIX fluorescence. Thus, our results demonstrated that PDD with 5-ALA has substantial clinical potential for GIST diagnosis.

## Introduction

GISTs are the most common submucosal tumors (SMTs) of the gastrointestinal tract observed in clinical practice, with a population prevalence of 14–20 cases per million [1, 2]. GISTs can occur anywhere in the gastrointestinal system, with 50%–60% located in the stomach, 30%–35% in the small intestine, 5% in the colon and rectum, and < 1% in the esophagus [3]. If the GIST is localized, the 5-year survival rate is 94%; when spread locally, this becomes 82%, and if the GIST exhibits distant metastasis at the time of diagnosis, the 5-year survival rate is 52% [4]. SMTs are derived from mesenchymal cells, and because of this, SMTs are generally enveloped by normal mucosa, which makes it difficult to differentially diagnose GISTs from other types of SMTs using conventional endoscopy. At present, endoscopic ultrasound-guided fine needle aspiration (EUS-FNA) has been developed, which enables pathological diagnosis of SMTs, including GISTs [5]. However, such diagnosis requires advanced technical skills that are limited to expert endoscopists. Additionally, performing EUS-FNA targeting small SMTs < 2 cm in diameter is especially difficult. GISTs occur anywhere along the gastrointestinal system and have malignant potential. Moreover, no effective treatment strategies, except for surgical resection, are currently available for GISTs. Hence, the present situation of diagnosis and treatment of GISTs is challenging.

**Fig 1:**
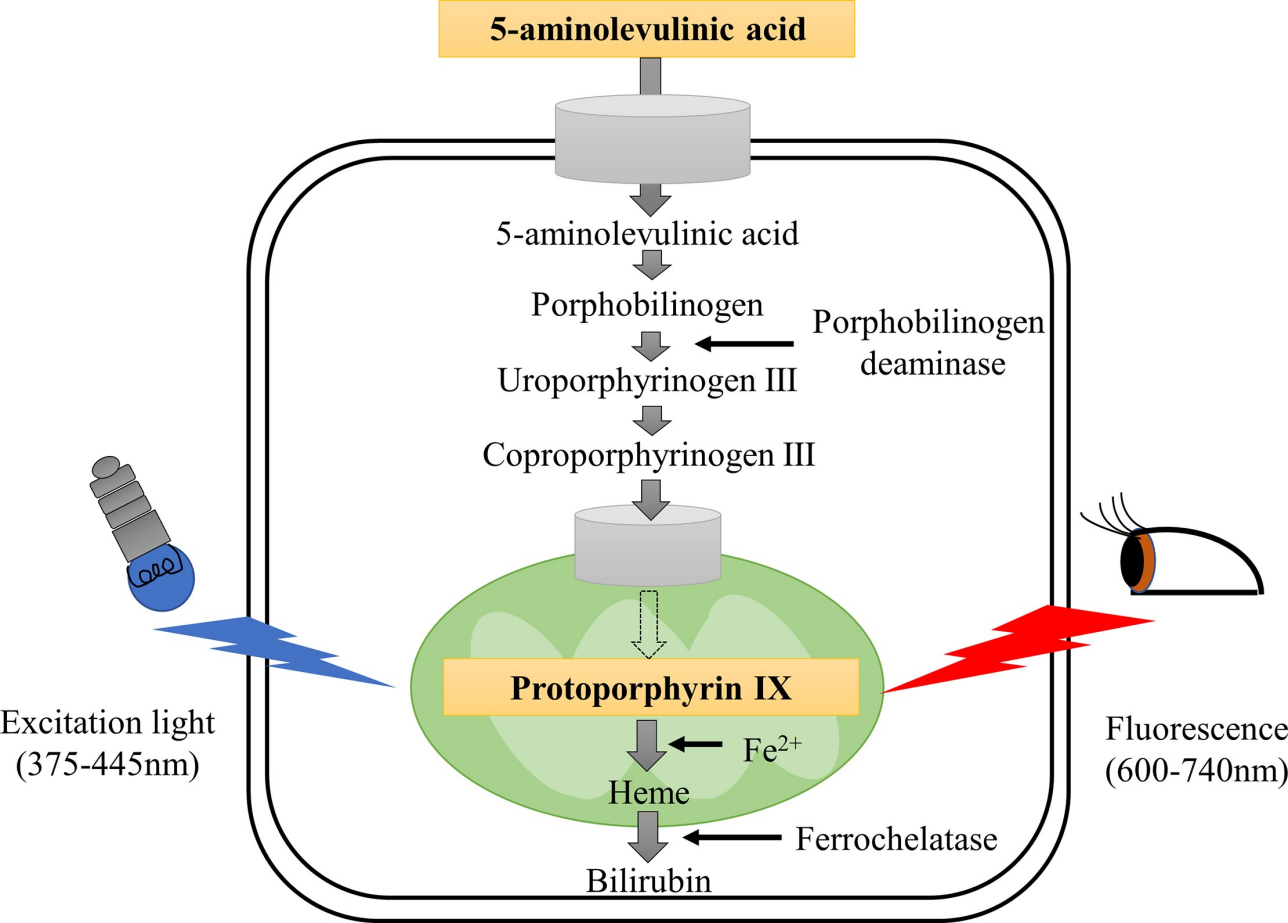
Schematic illustration of PpIX-metabolizing pathway and mechanism of PDD. 5-ALA is a metabolic precursor of heme in the heme synthesis pathway and is further metabolized into PpIX. PpIX emits red fluorescence (600–740 nm) when excited with blue-violet light (375–445 nm).

Photodynamic diagnosis (PDD), wherein tumor and normal tissues are identified by characteristically different fluorescent wavelengths, has been demonstrated to be an effective diagnostic technique for certain types of tumors, and is widely used in clinical applications [6]. PDD is based on the preferential uptake of photosensitizers by tumor tissues and its detection by fluorescence emission upon laser excitation to distinguish tumors from normal tissue. 5-aminolevulinic acid (5-ALA), a standard photosensitizer used in PDD, is a naturally occurring amino acid that has been widely used in clinical applications owing to its specificity toward some tumors and its demonstrated safety [7–11]. In Japan, 5-ALA PDD has been approved for health insurance coverage in cases of brain tumors (in particular, malignant glioma) and bladder tumors. 5-ALA is synthesized from glycine and succinyl-CoA by the enzyme 5-ALA synthase, which is dependent on vitamin B6 in mitochondria. 5-ALA is not itself fluorescent; however, it is a metabolic precursor in the heme biosynthetic pathway, and is further metabolized into endogenous fluorescent protoporphyrin IX (PpIX) (Fig 1) [12, 13]. PpIX emits red fluorescence between 600 and 740 nm when irradiated with 375–445 nm laser illumination [14–16]. This conversion of 5-ALA to its active compound PpIX in tissues has been used for PDD in many clinical applications, particularly because it substantially accumulates in tumor cells rather than in normal cells [7–9, 17, 18]. The reasons why PpIX accumulates much more in cancer cells than in normal cells are unclear, but two major hypotheses have been proposed: (1) PpIX accumulates in cancer cells because of a decrease in ferrochelatase activity; (2) 5-ALA has a high affinity for cancer cells [12, 19, 20].

To the best of our knowledge, many reports have been published regarding the accumulation of PpIX in different types of human cancer cell lines [21–36], but PDD of GISTs using 5-ALA has not yet been reported. In a similar study concerning PDD, Fujimoto et al. reported that molecular fluorescent imaging using a near-infrared (NIR) photosensitizer conjugated anti c-kit antibody was able to detect GISTs, making NIR irradiation a very efficacious theranostic (a newly coined term combining “therapeutics” and “diagnosis”) technology for GIST [37]. We previously demonstrated the antitumor effects of photodynamic therapy (PDT) in GIST using glucose-conjugated chlorin [38, 39]; however, PDD of GIST using 5-ALA has not been reported to date. Therefore, we investigated whether PDD with 5-ALA could be used for GIST detection as this could have therapeutic potential in combination with glucose-conjugated chlorin. Here, we show that PDD with 5-ALA has clinical potential for GIST diagnosis.

## Materials and methods

### Ethics statement

This study was carried out in strict accordance with the recommendations and guidelines of the Nagoya City University for Animal Experiments. The protocol was approved by the Committee of Laboratory Animal Facility at Nagoya City University for Experimental Animal Science under permit number 19–024. All experiments involving animals were performed under anesthesia, and all efforts were made to minimize suffering.

### Photosensitizer

5-ALA was obtained from Cosmo Bio Co., Ltd. (Tokyo, Japan) and dissolved in 0.9% normal saline at a concentration of 720 mmol/L. For the *in vitro* studies, 5-ALA was diluted in cell culture medium.

### Cell line and culture methods

The human GIST cell line GIST-T1 (code number: GIST01, Cosmo Bio Co.) was purchased in 2014. In addition to negative test results for mycoplasma contamination, cell line authentication and characterization were performed by Cosmo Bio Co. The International Council for Laboratory Animal Science (ICLAS) Monitoring Center, Central Institute for Experimental Animals, tested the cell line for the presence of viruses, with negative results. All experiments using cells in this study were performed for less than 20 passages. GIST-T1 cells were cultured in high glucose (4500 mg/L) Dulbecco’s modified Eagle’s medium (DMEM, Wako Pure Chemical Industries, Osaka, Japan) supplemented with 10% heat-inactivated fetal bovine serum (Biosera, Nuaillé, France) and 1% penicillin-streptomycin-amphotericin B suspension (Wako Pure Chemical Industries) under 5% CO_2_ and at 37°C.

### Flow cytometric analysis

GIST-T1 cells were resuspended in 6-cm culture dishes at a density of 1 × 10^6^ cells/well in culture medium and incubated at 37°C with 5% CO_2_ for 24 h. Following this, the medium was replaced with fresh medium supplemented with 0.89 mmol/L 5-ALA added to the dishes for 0, 15, 30, and 60 min to evaluate the accumulation of photosensitizer in cells. After washing the cells with phosphate-buffered saline (PBS) three times, cells were removed from the culture dish after trypsin-EDTA treatment for analysis using a flow cytometer with excitation at 405 nm and emission at 680 nm. All flow cytometric examinations were performed on a FACSCanto II (BD Biosciences, San Jose, CA, USA), and 10,000 events were counted and analyzed using FlowJo software (BD Biosciences).

### Subcellular localization of 5-ALA

GIST-T1 cells were seeded into 8-well chamber plates at a density of 1 × 10^4^ cells/well and incubated at 37°C with 5% CO_2_ for 24 h. Subsequently, 5-ALA was added to the culture media to a final concentration of 8 mmol/L, and the cells were further incubated for 2 h for staining with organelle-specific fluorescent probes. Lysosomes were stained with 0.1 μmol/L LysoTracker Green (Thermo Fisher Scientific, Waltham, MA, USA) at 37°C with 5% CO_2_ for 30 min, mitochondria with 0.1 μmol/L MitoTracker Green FM (Thermo Fisher Scientific) at 37°C with 5% CO_2_ for 10 min, Golgi with 5 μmol/L NBD C6-ceramide (Thermo Fisher Scientific) at 4°C on ice for 30 min, and endoplasmic reticulum (ER) with 0.1 μmol/L ER-Tracker Green (Thermo Fisher Scientific) at 37°C with 5% CO_2_ for 30 min. After incubation under each condition, the culture media were replaced with fresh media to remove free dyes. Stained cells were directly examined for accumulation in mitochondria, followed by fixation with 4% paraformaldehyde for accumulation identification in lysosomes, Golgi, and ER. Intracellular localization was visualized using a confocal laser microscope (FV3000, Olympus, Tokyo, Japan) and cellSens imaging software (Olympus). Band-pass emission filters of 500–540 nm and 488 nm for organelle-specific fluorescent probes, and 570–670 nm and 561 nm for PpIX were used. We measured the fluorescence intensity profiles. Arrows in micrographs showed the direction of scanning, and the histograms showed the strength of the fluorescent signals for evaluation of the peaks and trends of each luminescence. Co-localization was measured the whole each image, and the average values were calculated. Fluorescence intensity profiles were examined via confocal imaging, and quantitative analysis was performed using cellSens imaging software. Data from three independent experiments are presented as means ± standard deviation (SD). Statistical significance was determined by Tukey’s multiple comparison test and was set at P < 0.01.

### Animal models

Our experiments used murine animal models involving pathogen-free female nude mice (BALB/c Slc-nu/nu), 6–8 weeks of age, with a body weight of 18–22 g, which were purchased from Shizuoka Laboratory Animal Center (Shizuoka, Japan). Fifty mice were used in this study, including in preliminary experiments. Mice were kept under pathogen-free conditions under controlled temperature and humidity, 12 h light and 12 h dark cycle conditions, and fed a sterilized pellet diet with water *ad libitum*. Before commencing any intervention, all mice were acclimatized for at least 2 weeks in our animal facility. The health of the mice was monitored, and tumor sizes were measured every 2 days. Tumor volumes were calculated using the following formula: volume (mm^3^) = length (mm) × width (mm) × height (mm) × 0.5. Animals found to be unhealthy (loss of ambulation, appetite, or weight) were promptly planned to be euthanized. In addition, any mouse with a tumor size of 1000 mm^3^ was designated to be euthanized. None of the mice were found to be unhealthy or died throughout this study. Cervical dislocation was used for euthanasia. Mice were anesthetized by intraperitoneal injection of ketamine (100 mg/kg) and xylazine (10 mg/kg) reconstituted in physiological saline solution. Xenograft models of flank tumors were established by subcutaneously implanting 3.4 × 10^7^ GIST-T1 cells in 200 μL of medium and equivalent amounts of Matrigel (Corning, New York, USA) under the flanks of mice. Xenograft models of peritoneal dissemination were established by implanting 2.5 × 10^7^ GIST-T1 cells in 100 μL of medium into the abdominal cavity. PDD was performed after tumor size reached 100–300 mm^3^ in flank tumor models, and all mice were euthanized at the time of PDD.

### *In vivo* PDD

*In vivo* PDD was started when tumor sizes reached 100–300 mm^3^ in flank tumor models 5 weeks after the injection of tumor cells. We considered three groups (n = 3) of flank tumor models as follows: a dose of 300 mg/kg 5-ALA was injected and PDD conducted after 4 h, 300 mg/kg and 7 h, and 600 mg/kg and 4 h. Peritoneal seeding models were intended to form peritoneal dissemination nodules around the same time as the flank tumor was established, and a dose of 300 mg/kg 5-ALA was injected via the tail vein. Mice were sacrificed after 4 and 7 h, in accordance with our institutional guidelines, followed by tumor extraction. Tumors were exposed to white light and LED illumination (420 nm). Images were captured using a high-resolution camera equipped with an optical filter (cut-off 470 nm, long band pass filter VIS 470 nm, (Asahi Spectra, Tokyo, Japan)).

### Spectrophotometric analysis

We examined the accumulation of photosensitizers in murine xenograft tumors using a semiconductor laser with a VLD-M1 spectrometer (M&M, Tokyo, Japan) and its accessory software (BW-Spec V3. 24; B&W TEK, Inc., Newark, DE, USA). The spectrometer emitted laser light with a peak wavelength of 405 ± 1 nm and a light output of 140 mW. We used the manufacturer’s software to analyze spectral waveforms. The relative fluorescence intensity ratio in tumor and normal tissues was calculated by dividing the relative fluorescence intensity of PpIX (635 nm) by that of autofluorescence (505 nm) [38–41]. Following injection of 300 mg/kg 5-ALA in the xenograft flank tumor model animals after 4 h, the data—in view of the results of *in vivo* PDD—was obtained when conditions were optimal. Data from three independent experiments are presented as means ± standard deviation (SD). Significance was calculated using Student’s *t-*test.

### Statistical analysis

Descriptive statistics and samples were analyzed using Prism software (v.6.0; GraphPad Software, Inc., San Diego, CA, USA). Statistical significance was determined by Tukey’s multiple comparison test for quantitative analysis of subcellular localization of PpIX. Student’s *t*-test was performed for spectrometric analyses.

## Results

### PpIX accumulated in GIST-T1 cells

**Fig 2:**
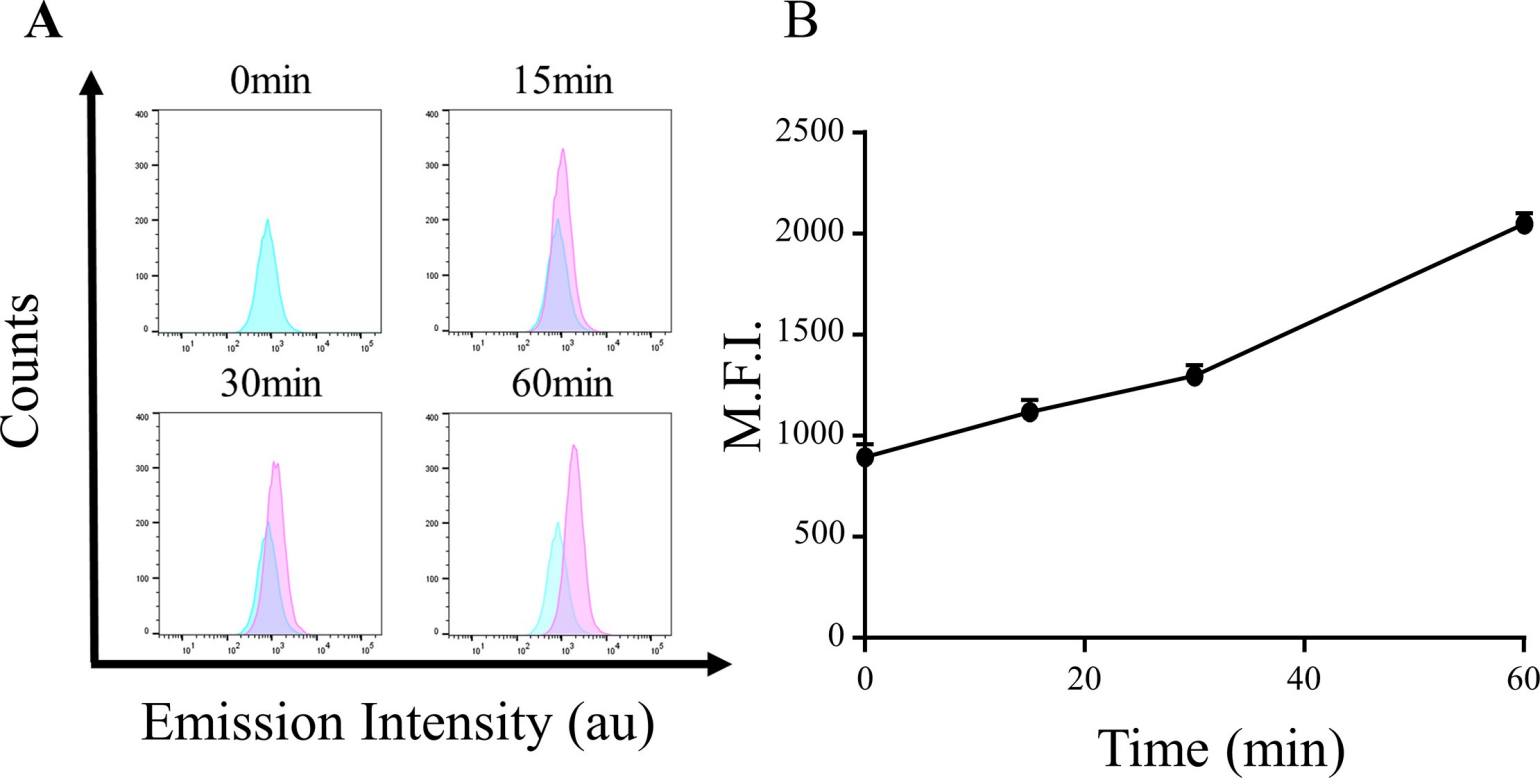
PpIX accumulation in GIST-T1 cells *in vitro*. GIST-T1 cells were loaded with PpIX for different durations. Accumulation of PpIX within cells was analyzed using FACS with excitation at 405 nm and emission at 680 nm. (A) Histogram data from FACS. The abscissa (X-axis) indicates intensity of emission and the ordinate (Y-axis) represents number of cells. The green and red histograms represent cell samples at 0 min (as a control) and cell samples at each indicated time point, respectively. (B) Temporal changes in mean fluorescence intensity (M.F.I.). The abscissa (X-axis) indicates time and the ordinate (Y-axis) of the graph represents M.F.I.

We examined the uptake of PpIX by GIST-T1 cells *in vitro*. We incubated cells with 0.89 mmol/L 5-ALA for 0, 15, 30, and 60 min. Then, we measured the intensity of the characteristic red fluorescence at the single-cell level using fluorescence-activated cell sorting (FACS). The accumulation of PpIX in GIST-T1 cells increased in a time-dependent manner (Fig 2).

### PpIX in GIST-T1 cells accumulates mainly in lysosomes

**Fig 3:**
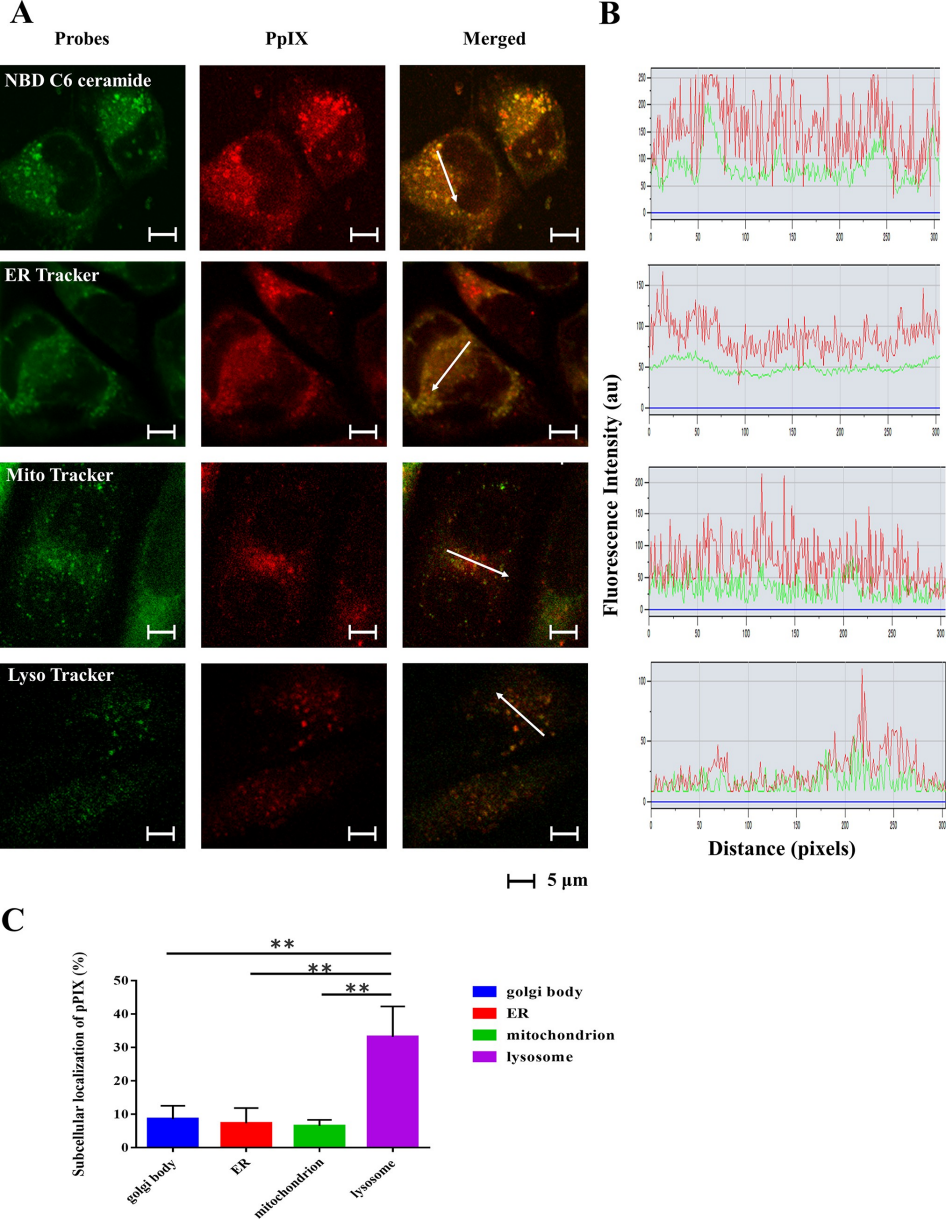
Subcellular localization of PpIX. GIST-T1 cells were loaded with 5-ALA (8 mmol/L) for 2 h and stained with MitoTracker Green, LysoTracker Green, NBD-C6 ceramide Green, or ER-Tracker Green. Intracellular localization was visualized by confocal microscopy (original magnification, × 350; scale bar, 5 μm). (A) Each row represents the fluorescence of the organelle-specific probes and PpIX, respectively. (B) The fluorescence intensity profiles of PpIX (red lines) and the organelle probes (green lines) were examined along the arrows in the confocal images. (C) Quantitative analysis of subcellular localization of PpIX. Statistical significance was determined using Tukey’s multiple comparison test and was set at **P < 0.01. The rate of concordance of PpIX levels to each organelle probe was significantly higher in lysosomes (P < 0.01).

Accumulation and subcellular localization of PpIX were investigated using confocal microscopy employing fluorescent probes to specifically label intracellular organelles. Cells were loaded with 5-ALA and incubated with each organelle-specific fluorescent probe (MitoTracker Green, LysoTracker Green, NBD C6 ceramide Green, or ERTracker Green) to label mitochondria, lysosomes, Golgi, and ER, respectively. The corresponding detection of PpIX with LysoTracker indicated accumulation in lysosomes (Fig 3A). The fluorescence intensity profiles for PpIX detection exhibited a tendency to correlate with the lysosome tracking marker (Fig 3B), and quantitative analysis revealed that the percentage of subcellular localization of PpIX was significantly greater in lysosomes (P < 0.01).

### PDD with 5-ALA distinctly identifies GIST *in vivo*

**Fig 4:**
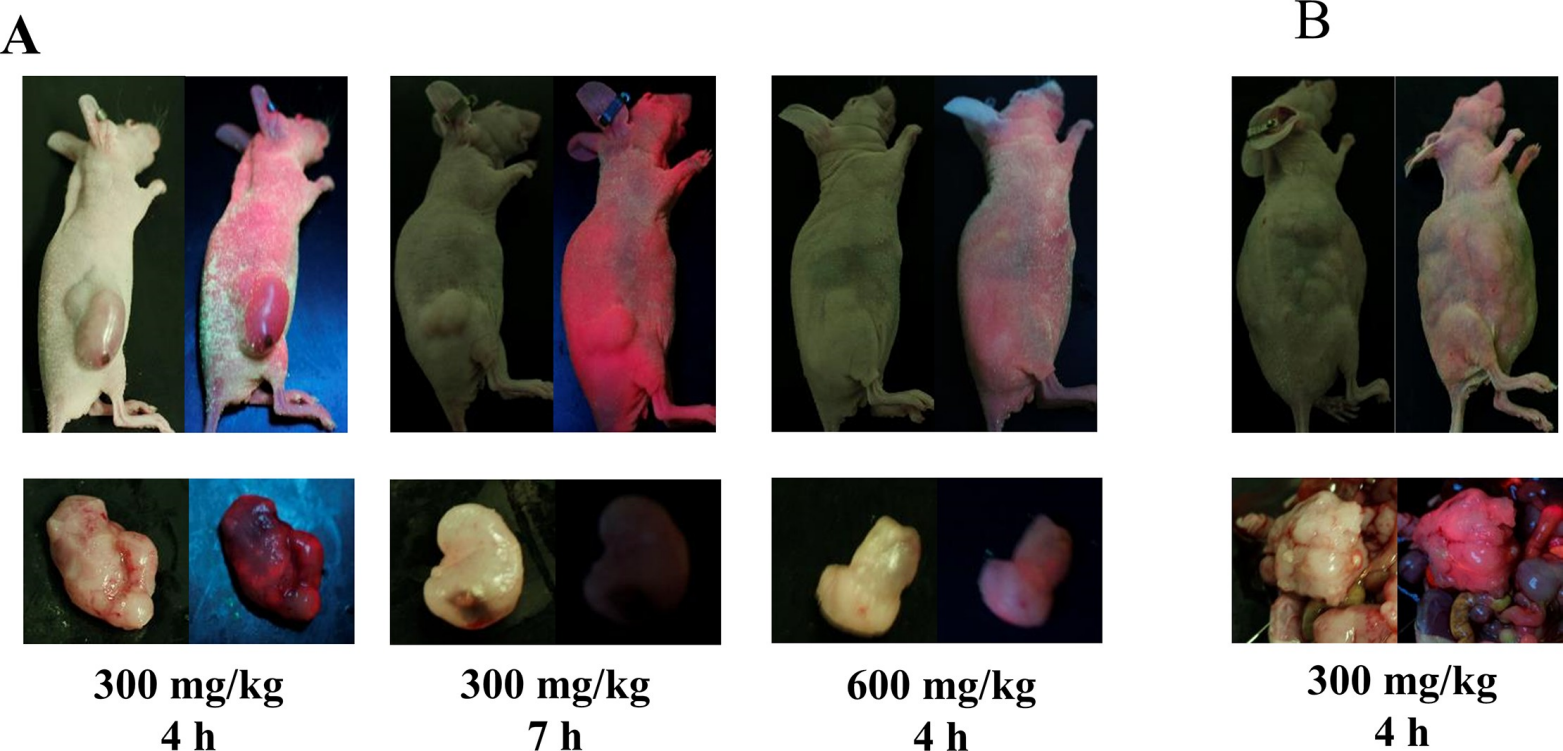
Fluorescent imaging of GIST xenograft models. Images were captured using a high-resolution camera equipped with an optical filter. Each image was photographed under white light (left side) and LED illumination (right side). (A) Xenograft models of flank tumors. Strong fluorescence was observed in mice receiving 300 mg/kg 5-ALA for 4 h. (B) Xenograft models of peritoneal seeding tumors. Peritoneal dissemination could not be identified through skin via LED light, but strong fluorescence was observed in extracted tumors.

We tested photosensitizer fluorescence in xenograft tumor mouse models. After the implanted tumors grew to 100–300 mm^3^ in flank tumor models, 5-ALA was administered to mice, and all mice were euthanized after several hours. The extracted tumors were exposed to white light and LED irradiation. Tumors treated with 5-ALA clearly emitted red fluorescence under 420 nm LED excitation. In the flank tumor models, the highest level of fluorescence was measured at a dose of 300 mg/kg 5-ALA for 4 h. In peritoneal seeding models, we could not identify peritoneal dissemination through the skin via LED light, but the extracted tumors emitted strong fluorescence (Fig 4).

### PpIX accumulation in xenograft tumors

**Fig 5:**
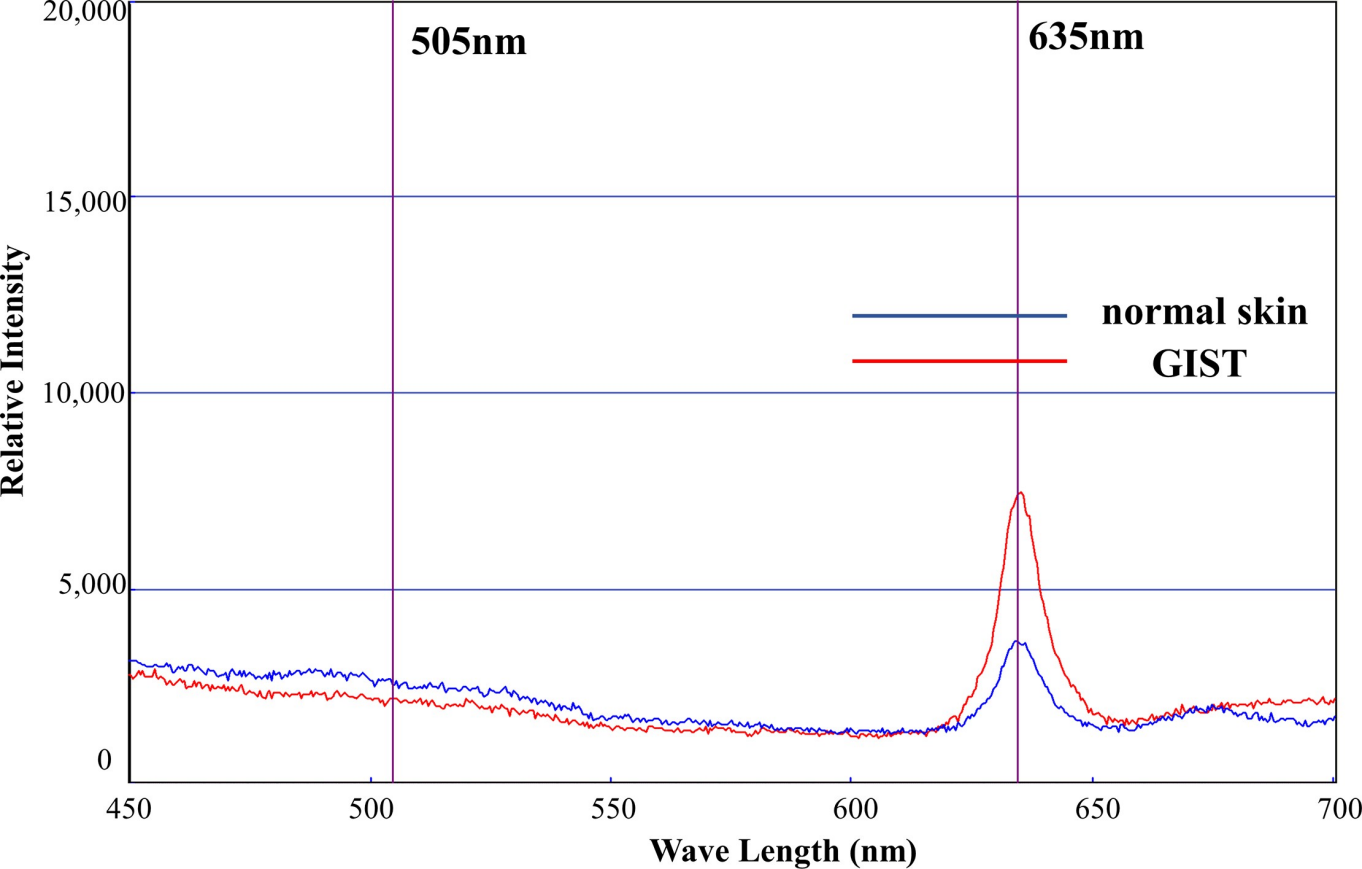
5-ALA accumulation in GIST *in vivo*. Flank tumor models were administered with 300 mg/kg 5-ALA via the tail vein. Four hours after 5-ALA administration, the spectral waveforms of normal skin tissue (blue line) and GIST (red line) were examined using a VLD-M1 spectrometer. A peak of fluorescence emission was observed at 635 nm, corresponding to PpIX.

We examined PpIX accumulation in xenograft tumors established by subcutaneous or intraperitoneal implantation of GIST-T1 cells. After intravenous injection (via the tail vein) of 5-ALA, the spectral waveforms of xenograft tumors were measured using a spectrometer (VLD-M1). Spectral waveform analysis revealed that a peak of fluorescence emission was observed at 635 nm, corresponding to PpIX (Fig 5). Generally, autofluorescence levels were centered around 505 nm. We then examined relative fluorescence intensity ratios (at 635/505 nm) of photosensitizers in tumors and normal tissues surrounding the tumors using a spectrometer. Data were obtained from three independent experiments in each group. The increase in the ratio demonstrated the significance of the difference in accumulation between normal skin and GIST (P < 0.05) (Table 1).

**Table 1:** Autofluorescence in normal skin tissues and fluorescence intensity using 5-ALA in GIST.

	Normal skin	GIST	*P*-value
**Emission at 505 nm**	1960 ± 260	2080 ± 200	0.353
**Emission at 635 nm**	2210 ± 960	7110 ± 600	0.011 < 0.05
**635 nm / 505 nm ratio**	1.09 ± 0.34	3.43 ± 0.05	0.012 < 0.05

Autofluorescence in normal skin tissues and fluorescence intensity following treatment with 5-ALA in GIST and relative fluorescence intensities. The conditions were as described in the legend of Fig 5. These data were obtained from xenograft flank tumor models injected with 300 mg/kg 5-ALA after 4 h.

## Discussion

In this study, we observed excellent *in vitro* and *in vivo* accumulation and fluorescence using 5-ALA in GISTs. 5-ALA is a standard photosensitizer used worldwide for PDD [42]. Therefore, we investigated the potential of PDD in GIST by examining the *in vitro* and *in vivo* accumulation of PpIX, which is a metabolite of 5-ALA.

We observed that *in vitro* accumulation of PpIX was mainly localized to tumor cell lysosomes, which have been proposed to be critical for photosensitizer action [43]. Hamblin et al. reported that photosensitizers principally accumulate in endosomes and lysosomes, unless they are metabolized and released from lysosomes [44]. In several cancer cell types, the synthesis of PpIX is typically promoted by an increase in the activity of the β-transporter and porphobilinogen deaminase, whereas the metabolizing pathway of PpIX is inhibited by an increase in the activity of the transferrin receptor and a decrease in the activity of ferrochelatase (Fig 1). Moreover, the requirement for heme is low in cancer cells because such cells depend on glycolytic energy production (the so-called Warburg effect) [45]. As a result, PpIX accumulates in cancer cells [46, 47]. These reports confirm our result that PpIX accumulation in GIST-T1 cells was mainly localized to lysosomes. We previously synthesized and reported PDD using oligosaccharide-conjugated chlorin (O-chlorin) [48]. In our previous study, we confirmed that O-chlorin accumulated in MKN45 (a human gastric cancer cell line), and was mainly localized to lysosomes. In addition, another photosensitizer, hypericin, is reported to localize to the ER and lysosomes in HeLa cells [49]. Based on these reports, we consider that many photosensitizers used in the field of PDD tend to accumulate in lysosomes. On the contrary, certain photosensitizers used for PDT accumulate in various subcellular compartments, such as photolon accumulation in mitochondria, lysosomes, and Golgi apparatus [50], and photosensitizer I, which is a novel photosensitizer reported by Peng-Xi Li, accumulates in the mitochondria and ER of HeLa cells [51]. A recent study showed that the same photosensitizers exhibit different localizations in various cell lines [52]. Therefore, identification of photosensitizer localization requires further consideration.

Fluorescence emission was observed in murine xenograft tumors treated with 5-ALA. However, this emission was dependent on the time elapsed following 5-ALA administration, as well as its concentration. Higher doses of photosensitizer rendered the visual distinction of fluorescence emitted by the tumor and normal tissue difficult, thereby leading to incorrect PDD of SMTs. Similarly, clear fluorescence was observed only within a specific timeframe following 5-ALA administration. In previous studies, the optimal time to evaluate the fluorescence of 5-ALA in PDD was indicated to be 1–4 h [51, 53]. Although these studies involved different cell lines and routes of drug administration, they do not contradict our results.

Extensive research has been conducted concerning malignancies of the digestive tract, including gastric, esophageal, and colon cancers, among others. In Japan, peritoneal metastasis of gastric cancer has been a major area of focus for the development of pharmaceutical agents. Laser-based photodynamic endoscopic diagnosis of early gastric and colorectal cancers using 5-ALA has been reported, although the issue of fluorescent intensity differences among histopathological types has not yet been resolved [15, 54]. However, a recent study suggested that gastric cancer cell lines expressing high levels of ATP-binding cassette transporters tend to exhibit low levels of 5-ALA staining [55]. We previously demonstrated the efficacy of PDT with a novel glucose-conjugated photosensitizer, glycocon-conjugated chlorin (5, 10, 15, 20-tetrakis [4- [β-D-glucopyranosylthio- 2, 3, 5, 6-tetrafluorophenyl]-2, 3, [methano [N-methyl] iminomethano] chlorin, H2TFPC-SGlc) in GIST, both *in vitro* and *in vivo* [38]. Nevertheless, the GIST diagnostic approach presents a greater potential for improvement compared to conventional methods.

In summary, the accumulation of PpIX in GIST-T1 cells, specifically in lysosomes, as well as the photodynamic detection of PpIX in murine xenograft models, demonstrates a strong potential for PDD and clinical applications in the future.

## Supporting information

S1 Fig Fluorescent imaging of murine GIST xenograft models. Images were acquired using a high-resolution camera equipped with an optical filter. Each image was photographed under white (left side) and LED light illumination (right side). The remaining two mice of each group, except for those shown in Fig 4, are shown in this figure. Mice used during preliminary experiments under other conditions (600 mg/kg 5-ALA for 7 h and control) are also included.
(TIF)
