# Supplementary figures and images for: 5-aminolaevulinic acid (5-ALA) accumulates in GIST-T1 cells and photodynamic diagnosis using 5-ALA identifies gastrointestinal stromal tumors (GISTs) in xenograft tumor models
Source: PLoS One. 2021 Apr 7;16(4):e0249650. doi: 10.1371/journal.pone.0249650 (PMC8026038; doi:10.1371/journal.pone.0249650)

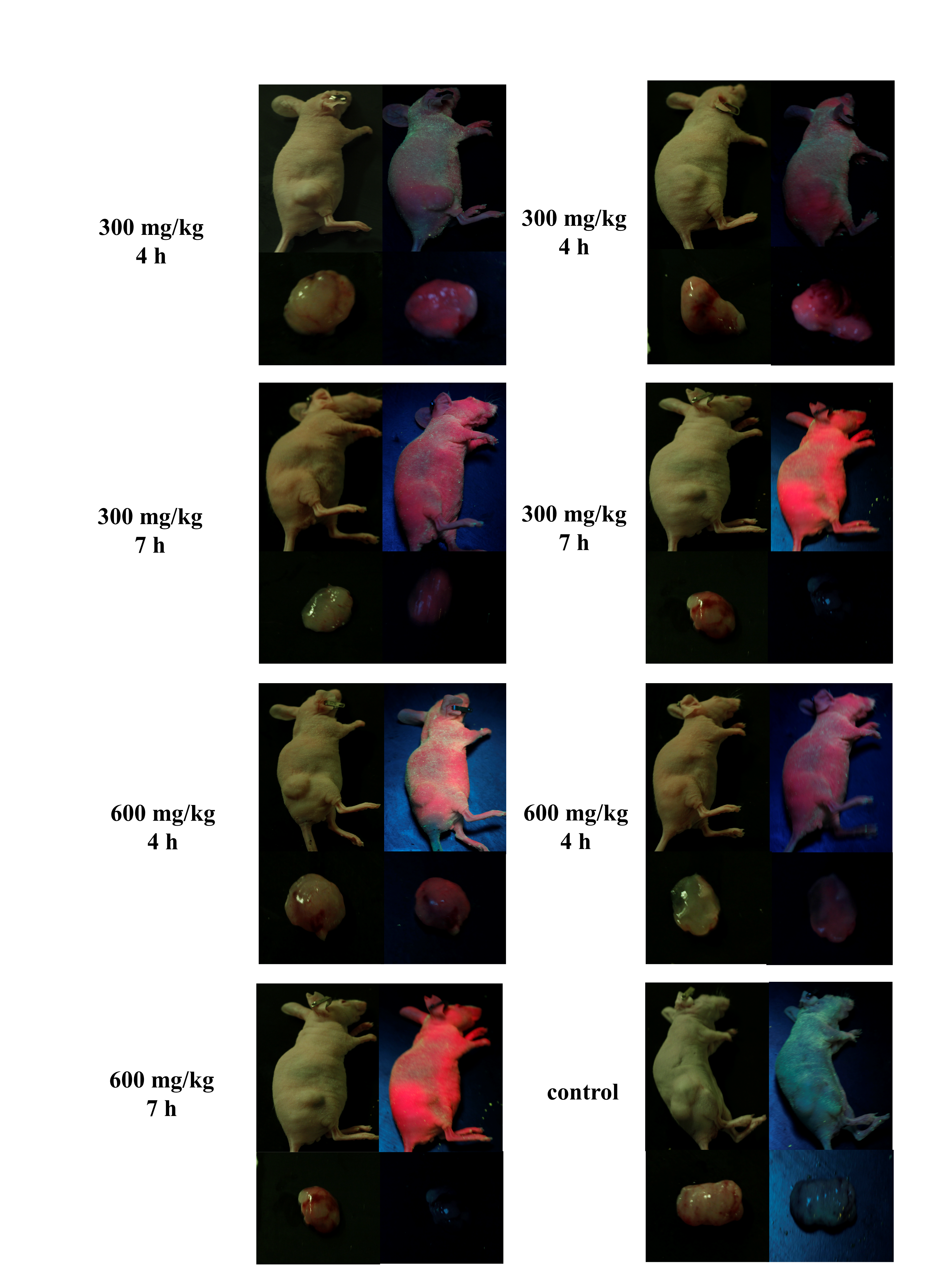

Supplement: S1 Fig — Images were acquired using a high-resolution camera equipped with an optical filter. Each image was photographed under white (left side) and LED light illumination (right side). The remaining two mice of each group, except for those shown in Fig 4, are shown in this figure. Mice used during preliminary experiments under other conditions (600 mg/kg 5-ALA for 7 h and control) are also included. (TIF) [file pone.0249650.s001.tif]
